# Hemoglobin glycation index as a useful predictor of therapeutic responses to dipeptidyl peptidase-4 inhibitors in patients with type 2 diabetes

**DOI:** 10.1371/journal.pone.0171753

**Published:** 2017-02-09

**Authors:** Yu-Wei Chen, Jun-Sing Wang, Wayne H-H Sheu, Shih-Yi Lin, I-Te Lee, Yuh-Min Song, Chia-Po Fu, Chia-Lin Lee

**Affiliations:** 1 Division of Endocrinology and Metabolism, Department of Internal Medicine, Taichung Veterans General Hospital, Taichung, Taiwan; 2 School of Medicine, National Yang Ming University, Taipei, Taiwan; 3 College of Medicine, National Defense Medical Center, Taipei, Taiwan; 4 Center for Geriatrics and Gerontology, Taichung Veterans General Hospital, Taichung, Taiwan; 5 Graduate Institute of Biomedical Electronics and Bioinformatics, College of Electrical Engineering and Computer Science, National Taiwan University, Taipei, Taiwan; 6 Department of Public Health, College of Public Health, China Medical University, Taichung, Taiwan; 7 Department of Medical Research, Taichung Veterans General Hospital, Taichung, Taiwan; Weill Cornell Medical College Qatar, QATAR

## Abstract

**Introduction:**

A high hemoglobin glycation index (HGI) and glycated hemoglobin (HbA1c) level are associated with greater inflammatory status, and dipeptidyl peptidase-4 (DPP-4) inhibitors can suppress inflammation. We aimed to evaluate the relationship between HGI and the therapeutic effect of DPP-4 inhibitors.

**Methods:**

This retrospective cohort study followed 468 patients with type 2 diabetes receiving DPP-4 inhibitor treatment for 1 year. Estimated HbA1c was calculated using a linear regression equation derived from another 2969 randomly extracted patients with type 2 diabetes based on fasting plasma glucose (FPG) level. The subjects were divided into two groups based on HGI (HGI = observed HbA1c - estimated HbA1c). Mixed model repeated measures were used to compare the treatment efficacy after 1 year in patients with a low (HGI<0, n = 199) and high HGI (HGI≧0, n = 269).

**Results:**

There were no significant group differences in mean changes of FPG after 1 year (-12.8 and -13.4 mg/dL in the low and high HGI groups, respectively). However, the patients with a high HGI had a significantly greater reduction in HbA1c from baseline compared to those with a low HGI (-1.9 versus -0.3% [-20.8 versus -3.3 mmol/mol]). Improvements in glycemic control were statistically significantly associated with the tested DPP-4 inhibitors in the high HGI group (-2.4, -1.4, -1.2 and -2.2% [-26.2, -15.3, -13.1 and -24.0 mmol/mol] for vildagliptin, linagliptin, saxagliptin and sitagliptin, respectively) but not in the low HGI group.

**Conclusions:**

The HGI index derived from FPG and HbA1c may be able to identify who will have a better response to DPP-4 inhibitors.

## Introduction

The prevalence of type 2 diabetes mellitus is increasing worldwide, especially in Asian countries, and this is a major challenge for health care systems [1]. The current management of diabetes aims to lower the glycosylated hemoglobin (HbA1c) level to decrease the risk of diabetes complications. However, the HbA1c level varies considerably even in individuals who have similar preceding mean blood glucose (MBG) levels. Between-person biological variations in HbA1c have been studied in healthy individuals without diabetes [2–4], and it has been suggested that between-patient differences in HbA1c are greater than within-subject variations in HbA1c, and that there is a tendency for some individuals to have persistently higher or lower HbA1c levels than expected [3]. Several investigators have also identified this phenomenon in patients with type 1 and type 2 diabetes using the hemoglobin glycation index (HGI) [5, 6]. The HGI is calculated as the difference between an individual’s observed HbA1c and estimated HbA1c (HGI = observed HbA1c - estimated HbA1c). An estimated HbA1c level is calculated from a linear regression equation and describes the relationship between HbA1c and blood glucose by including observed MBG or fasting plasma glucose (FPG) into the equation [5–7].

It is possible that less well controlled diabetic patients do not develop chronic diabetes complications, and vice versa. In this regard, both HbA1c and HGI have been proposed to be possible predictors of chronic complications of diabetes. Compared with a low HGI, diabetic patients with a high HGI have been shown to be prone to developing long-term complications of diabetes [8, 9]. Moreover, individuals with a high HGI were found to be associated with higher levels of inflammatory markers in a non-diabetic population [10], and inflammation has been reported to be a possible mechanism causing microvascular and macrovascular complications in diabetic patients [11, 12]. In addition, inflammation has been reported to play a role in insulin resistance and β-cell dysfunction [13], and therapies aimed at ameliorating inflammatory processes have been showed to improve insulin sensitivity and secretion in patients with diabetes [14]. Numerous recent clinical trials have shown evidence of improved glycemic control with treatment of dipeptidyl peptidase-4 (DPP-4) inhibitors [15, 16]. Of note, anagliptin was recently shown to ameliorates inflammation in vitro as well as in lipopolysaccharide-infused mice [17]. In addition, clinical studies have reported that oxidative stress and circulating inflammatory markers can be reduced by sitagliptin and vildagliptin in patients with type 2 diabetes [18–20]. The use of DPP-4 inhibitors has increased steadily since their introduction [21, 22], however their ability to decrease HbA1c level has shown variable results with different or even the same type of DPP-4 inhibitor, either as add-on therapy or monotherapy, with mean changes in HbA1c ranging from -0.24 to -1.4% (-2.6 to -15.3 mmol/mol) [23–25].

Based on the potential anti-inflammatory effects of DPP-4 inhibitors and as individuals with a high HGI have a higher inflammatory status, we hypothesized that diabetic patients receiving DPP-4 inhibitors may exhibit diverse therapeutic outcomes associated with the initial HGI status. This study aimed to examine this hypothesis.

## Materials and methods

### Study design and patient populations

This retrospective cohort study was performed at a medical center in central Taiwan. Patients receiving DPP-4 inhibitor treatment with type 2 diabetes were recruited from our outpatient clinic between July 2008 and June 2014. Their medical records and clinical parameters were reviewed. Patients’ records were de-identified and analyzed anonymously. Before the initiation of DPP-4 inhibitor therapy, the patients were either treatment naïve or had previously received oral anti-diabetic drugs (OADs). The patients were followed for 1 year from the first DPP-4 inhibitor prescription. During follow-up, no new OADs were added or changed to another type of DPP-4 inhibitor. The exclusion criteria were: (1) type 1 diabetes; (2) age under 18 years; (3) missing data for calculating the HGI; (4) past history that could interfere with erythrocyte life span (i.e. anemia, chronic renal failure, pregnancy). This study was approved by the Human Research Review Committee of Taichung Veterans General Hospital. The baseline variables of the study subjects are shown in Table 1. FPG and HbA1c were checked at 3-month intervals. HbA1c was measured using boronate affinity high-performance liquid chromatography (Premier Hb9210, Trinity Biotech, Ireland), and FPG was measured using either the hexokinase method (Labospect 008, Hitachi, High-Tech Co., Japan) or glucose oxidase method (A&T Glucose Analyzer GA05, A&T, Tokohama, Japan).

**Table 1:** Demographic and baseline characteristics of the study participant.

Variable	High HGI (n = 269)	Low HGI (n = 199)	P-value
Age, years	56±13	59±12	0.05
Male Sex, n (%)	154 (57.2)	114 (57.3)	1.00
Body weight (kg)	69±15	68±11	0.340
Body mass index (kg/m2)	26.2±4.4	25.8±3.5	0.415
HbA1c (%)	9.5±1.9 (80±21mmol/mol)	7.5±1.0 (58±11mmol/mol)	<0.001
FPG (mg/dL)	161±57 (8.9±3.1mmol/L)	160±46 (8.8±2.5mmol/L)	0.848
Creatinine (mg/dL)	0.9±0.4 (80±35μmol/L)	1.0±0.4 (88±35μmol/L)	0.161
Systolic BP (mmHg)	135±19	132±18	0.157
Diastolic BP (mmHg)	80±11	78±10	0.168
No. of glucose-lowering background drugs	1.5±0.9	1.4±0.8	0.489
Metformin, n (%)	173 (64.3)	129 (64.8)	0.922
Sulfonylurea, n (%)	146 (54.3)	98 (49.2)	0.304
Insulin, n (%)	20 (7.4)	4 (2.0)	0.01
Meglitinide, n (%)	8 (3.0)	3 (1.5)	0.368
AGI, n (%)	30 (11.2)	25 (12.6)	0.665
Thiazolidinedione, n (%)	36 (13.4)	25 (12.6)	0.890

Continuous variables are expressed as mean ± SD.

Categorical data are presented as numbers (percentages).

Abbreviations: AGI, α-glucosidase inhibitor; BP, blood pressure; FPG, fasting plasma glucose; HbA_1_c, glycated hemoglobin; HGI, hemoglobin glycation index.

### Calculation of the HGI and study endpoints

Most prior studies have calculated estimated HbA1c from MBG either from self-monitoring [5–7] or continuous glucose monitoring [26]. However, a previous study showed that HGI calculated from mean pre-breakfast glucose level was highly correlated with HGI derived from mean total glucose [5], and a statistical model to assess between-individual variations in HbA1c has been developed from FPG [9, 10]. We used a similar method to calculate the HGI. In brief, a linear relationship between FPG and HbA1c was developed from 2969 randomly selected patients with type 2 diabetes who visited our outpatient clinic. For each participant in this study, an estimated HbA1c level was calculated from the regression equation (HbA1c [%] = 4.6929+0.02071FPG [mg/dL], r2 = 0.3672) (To convert glucose to millimoles per liter, multiply by 0.0555). We then calculated HGI as HGI = observed HbA1c - estimated HbA1c. The patients were then assigned to low (HGI<0) or high HGI (HGI≧0) subgroups according to their baseline HGI values. The primary efficacy end points of this analysis were mean changes in HbA1c and FPG from baseline to 1 year.

### Statistical analysis

Continuous variables are reported as mean ± SD and categorical variables as numbers (percentages). Differences in clinical variables at baseline between HGI groups were tested with the independent t- test for continuous variables and the chi-square test for categorical variables. Changes in HbA1c and FPG from baseline to 1 year were tested using paired-t tests. Mixed model repeated measures (MMRM) was used to evaluate adjusted changes in annual HbA1c and FPG. Levels of HbA1c and FPG after 1 year were set as the dependent variable, and time was set as the independent variable. The adjusted mean changes in HbA1c and FPG from baseline to 1 year were defined as the β coefficients of time by MMRM. Changes in HbA1c and FPG between HGI groups and among different DPP-4 inhibitors were tested by interaction from MMRM. All statistical analyses were performed using the Statistical Package for Social Sciences software (IBM SPSS version 22.0; International Business Machines Corp, New York, USA).

## Results

A total of 468 (268 men) patients were analyzed. Baseline demographic, biochemical, and clinical characteristics as well as concomitant background therapies were compared between the two HGI groups (Table 1). Compared with the low HGI group, the high HGI group was younger with a higher initial mean HbA1c value but similar FPG level. In addition, the high HGI group was more likely to have used insulin before the start of the study.

**Fig 1:**
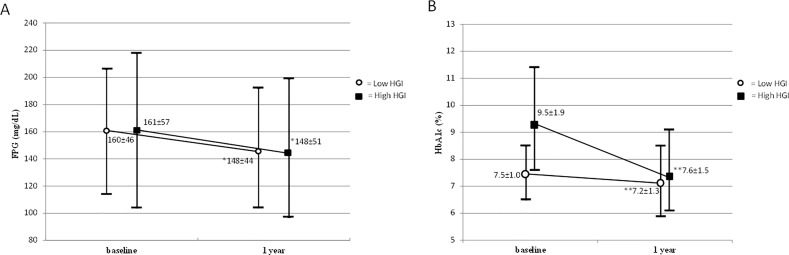
**Mean value from baseline to 1 year of dipeptidyl peptidase-4 inhibitor treatment in two groups according to baseline hemoglobin glycation index (HGI) in fasting plasma glucose (FPG) (*p* = 0.965) (A) and glycated hemoglobin (HbA**_**1**_**c) (*p* < 0.001) (B).** Data are presented as mean ± standard deviation (SD). * p = 0.002, **p<0.001 compared with baseline; where ‘*p*’ represents *p*-value of between-group difference. (To convert glucose to millimoles per liter, multiply by 0.0555.)

After 1 year of DPP-4 inhibitor treatment, the mean FPG level was significantly improved in both the high and low HGI groups (-13.4±69.5 mg/dL [-0.7±3.8 mmol/L] and -12.8±57.5 mg/dL [-0.7±3.2 mmol/L], respectively, p = 0.002 in both HGI groups), but with no significant between-group difference (p = 0.965) (Fig 1A). A similar pattern was observed with regards to the mean reduction in HbA1c level. The mean HbA1c level was significantly improved in both the high and low HGI groups (-1.9±2.3% [-21±25 mmol/mol] and -0.3±1.4% [-3±15 mmol/mol], respectively, p<0.001 in both groups). However, patients with a high HGI had a significantly greater reduction in HbA1c compared to those with a low HGI (p < 0.001) (Fig 1B).

As the initial HbA1c level would influence the absolute mean changes in HbA1c, the patients were further divided into tertiles according to baseline HbA1c level. The mean HbA1c was 7.0% (53 mmol/mol) for tertile 1 (range, < 7.7% [< 61 mmol/mol], n = 155), 8.2% (66 mmol/mol) for tertile 2 (range, 7.7–8.9% [61–74 mmol/mol], n = 159), and 10.7% (93 mmol/mol) for tertile 3 (range, > 8.9% [> 74 mmol/mol], n = 154). Across the tertiles there was a trend toward greater absolute mean changes in HbA1c from baseline in the higher tertiles (0.0%, -0.8% [-8.7 mmol/mol], and -2.9% [-31.7 mmol/mol] for tertile 1, tertile 2, and tertile 3, respectively). In addition, the patients with a high HGI had a greater reduction in HbA1c than those with a low HGI (-0.5% vs. 0.1% [-5.5 vs. 1.1 mmol/mol], -0.8% vs. -0.7% [-8.7 vs. -7.7 mmol/mol], and -3.0% vs. -2.2% [-32.8 vs. -24.0 mmol/mol] for tertile 1, tertile 2, and tertile 3, respectively) in each tertile. Changes in HbA1c were statistically different between the HGI subgroups in tertile 1 (p = 0.018) (data not shown).

**Fig 2:**
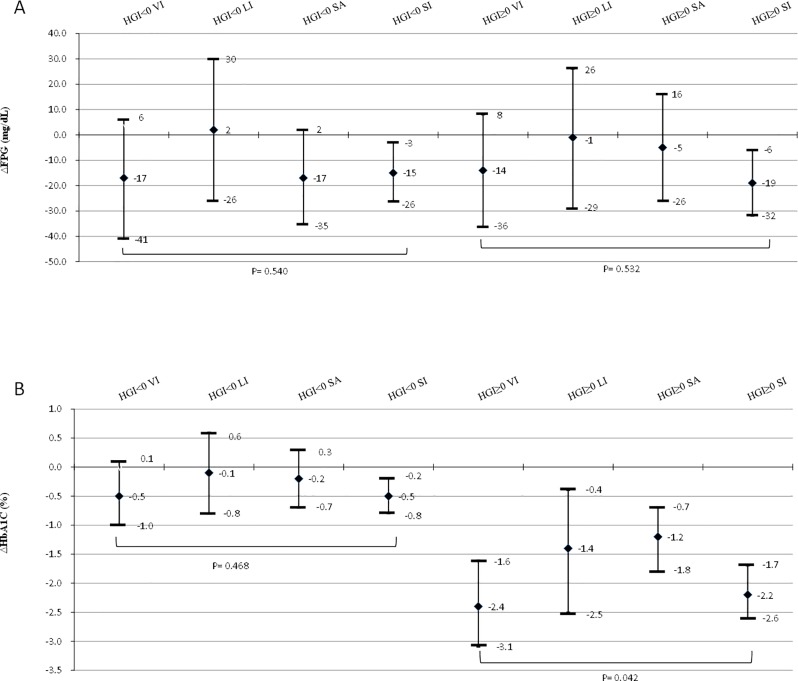
**Comparisons of the effect of different dipeptidyl peptidase-4 inhibitor treatment for 1 year on adjusted mean changes in fasting plasma glucose (FPG) (A) and glycated hemoglobin (HbA**_**1**_**c) (B) in the patients with a low and high hemoglobin glycation index (HGI).** Factors included in the analysis of variance statistical model were baseline oral anti-diabetes drugs, age, sex and renal function. VI = vildagliptin (n = 24 in the low HGI and n = 36 in the high HGI groups), LI = linagliptin (n = 33 in the low HGI and n = 31 in the high HGI groups), SA = saxagliptin (n = 45 in low HGI and n = 64 in the high HGI groups), SI = sitagliptin (n = 97 in the low HGI and n = 138 in the high HGI group). Error bars represent 95% confidence interval (CI). p-value for between-group difference. (To convert glucose to millimoles per liter, multiply by 0.0555)

We further explored the therapeutic efficacy among different types of DPP-4 inhibitors including vildagliptin, linagliptin, saxagliptin and sitagliptin. Alogliptin was not included as it was not available in our hospital. Important confounding factors for the therapeutic efficacy were considered including baseline oral anti-diabetes drugs, age, sex and renal function. After 1 year of DPP-4 inhibitor treatment, the adjusted mean changes in FPG from baseline in the low and high HGI groups were similar (-14, -1, -5, and -19 mg/dL [-0.8, -0.1, -0.3, and -1.0 mmol/L] for vildagliptin, linagliptin, saxagliptin and sitagliptin respectively in high HGI group; p for interaction = 0.532) (Fig 2A). Moreover, the adjusted mean changes in HbA1c from baseline were also not statistically significantly different in the low HGI group (-0.5, -0.1, -0.2, and -0.5% [-5.5, -1.1, -2.2, and -5.5 mmol/mol] for vildagliptin, linagliptin, saxagliptin and sitagliptin, respectively, p for interaction = 0.468) (Fig 2B). However, for the patients in the high HGI group, the adjusted mean changes in HbA1c were significantly greater in those taking vildagliptin or sitagliptin compared to those taking linagliptin or saxagliptin (-2.4, -1.4, -1.2, and -2.2% [-26.2, -15.3, -13.1, and -24.0 mmol/mol] for vildagliptin, linagliptin, saxagliptin and sitagliptin, respectively, p for interaction = 0.042) (Fig 2B).

## Discussion

Our results demonstrate that patients with type 2 diabetes with a high HGI managed by DPP-4 inhibitors had significant improvements in glycemic control compared with those with a low HGI. To the best of our knowledge, this is the first study to evaluate the efficacy of DPP-4 inhibitor treatment on glycemic control stratified by HGI. In this study, the patients with a high HGI were more likely to use insulin for sugar control, which may be because they tended to have a persistently high HbA1c level, which is consistent with previous studies [6, 9]. In addition, our finding of a markedly distinct HbA1c response in different HGI subgroups is also consistent with a former study, which suggested that baseline HbA1c was an important predictor of HbA1c response to DPP-4 inhibitors in patients with type 2 diabetes [27]. However, we divided our patients into tertiles according to baseline HbA1c level, and the results still showed between-subgroup differences in the HbA1c lowering effect, which may suggest that both baseline HbA1c and also HGI are major determinants of improvements in HbA1c.

Plasma glucose and HbA1c play a vital role in the complications of diabetes mellitus. However, there is evidence of between-individual differences in the relationship between plasma glucose and HbA1c [2–4, 26], and therefore the HGI is a useful tool to quantify the variability in this relationship [5, 6]. A previous study further investigated the clinical implication of HGI and found that it was associated with the risk of complications from diabetes. The Diabetes Control and Complications Trial evaluated the HGI and found that for patients with type 1 diabetes, those with a higher HGI tended to have greater risk of retinopathy and nephropathy [8]. In the Action to Control Cardiovascular Risk in Diabetes (ACCORD) trial, Hempe et al found that patients with a higher HGI were associated with a greater risk of hypoglycemia, and that intensive treatment for blood sugar failed to reduce the risk of cardiovascular end points and led to a higher mortality rate [9]. In our study analysis, relative to the patients with a low HGI, the decrease in HbA1c was clinically meaningful in the subjects with a high HGI treated with DPP-4 inhibitors. In addition, DPP-4 inhibitor treatment had a neutral effect on hypoglycemia, an important barrier in diabetes treatment. It could be a better choice for patients with a high HGI to safely and effectively reach their HbA1c target. Moreover, DPP-4 inhibitors have been shown to have favorable effects on inflammation and oxidative stress [18–20]. Taken together, these findings suggest that DPP-4 inhibitor treatment may be beneficial in individuals with a high HGI in terms of further reducing the risk of complications from diabetes.

Unlike prior studies which used mean blood glucose to calculated estimated HbA1c, we used FPG for three reasons. First, as noted above, previous studies have shown that HGI calculated from mean total glucose is highly correlated with using pre-breakfast glucose alone [5]. Second, in daily practice, many doctors use follow-up FPG and HbA1c measurements when the patients visit their clinic. In addition, for patients self-monitoring blood glucose at home, they prefer to use daily FPG, leading to a relative lack of data on daytime glucose levels to calculate MBG accurately. Third, previous studies have used FPG to calculate the HGI, and revealed that it is a useful tool to predict the clinical outcomes of diabetes, and the risks of hypoglycemia and inflammatory status [9, 10]. However, when using FPG to calculate the HGI, the relationship between HbA1c and FPG may be partially influenced by the daytime glucose level, as it was known that HbA1c was mainly contributed by FPG and post prandial glucose (PPG) [28–30]. The Pearson correlation coefficient in our study between HbA1c and FPG was 0.61. In meta-analysis, the pooled correlation coefficient between HbA1c and FPG was 0.61 [31]. This is almost the same with our data. In addition, the correlation coefficient between estimated and observed HbA1c in our study was 0.481 (S1 Fig). However, more strong correlation between observed and residual HbA1c (estimated HbA1c-observed HbA1c) was seen (r = 0.686) (S2 Fig), which is close to the finding of pooled coefficient between HbA1c and PPG in meta-analysis (r = 0.67) [31]. According to these findings, it suggested that HGI contained not only information of biological variation but also information of PPG. Due to inconvenient data of PPG in clinical practice, we used HGI calculated from FPG as an alternative index to predict the therapeutic response to DPP-4 inhibitor.

Our subgroup analysis found that patients receiving vildagliptin had greater adjusted mean changes in HbA1c from baseline compared with those receiving other DDP-4 inhibitors. The reason for this result is uncertain. It has been reported that improvements in the mean amplitude of glycemic excursions (MAGE) is associated with reductions in inflammatory markers and oxidative stress [18]. In addition, a previous study reported that a higher rate of MAGE was correlated with a higher HbA1c level [32], and that improvements in MAGE were accompanied with a reduction in HbA1c [33]. Former study compared vildagliptin and sitagliptin by using continuous glucose monitoring to evaluate the influence on blood glucose in patients with type 2 diabetes, and found that patients receiving vildagliptin had a significantly greater reduction in MAGE [18]. Similar findings were also reported in another study in which patients with type 2 diabetes receiving vildagliptin had a greater reduction in MAGE compared to those receiving saxagliptin [34]. Thus, as we calculated the HGI from FPG, our findings may partially be explained by vildagliptin resulting in greater reductions in MAGE thereby resulting in greater improvements in HbA1c.

There are several limitations to this study. First, this was a retrospective study. By reviewing the medical records, we could accurately check the prescriptions but not the patients’ compliance, which could have resulted in underestimating the drug efficacy, especially for those taken twice daily. However, our results are real world clinical results, and different study designs also have inherent limitations. Second, we only analyzed 1 year of DPP-4 inhibitor treatment due to a lack of data thereafter. However, previous studies have shown that the effects of DPP-4 inhibitors on HbA1c in patients with type 2 diabetes are highest in the first year, and then begin to decline from the second year of treatment [35, 36]. However, with a longer duration of diabetes, the decline in β-cell function is an important issue, and additional studies including long-term follow-up are needed. Finally, this study included a relatively small number of patients at a single institution, and the majority of the recruited patients received sitagliptin treatment as sitagliptin was the first approved agent at our hospital. Further studies are required to evaluate the long-term glucose lowering effects in a larger number of patients.

## Conclusion

In summary, we found that the administration of DPP-4 inhibitors in patients with a high HGI resulted in a significant reduction in HbA1c after 1 year of follow-up. This supports that the choice of diabetes therapy should be individualized. When starting therapy with a DPP-4 inhibitor, the HGI may help to quantify individual differences in daily clinical practice. Additional studies are needed to verify the results of this study and further investigate the use of HGI in other anti-diabetes medications.

## Supporting information

S1 Fig Scatter plot of correlation between estimated HbA1c and observed HbA1c. (TIF)

S2 Fig Scatter plot of correlation between residual value and observed HbA1c. Residual value = estimated value of HbA1c (%) minus observed value of HbA1c (%)
(TIF)
