# Supplementary figures and images for: Hemoglobin glycation index as a useful predictor of therapeutic responses to dipeptidyl peptidase-4 inhibitors in patients with type 2 diabetes
Source: PLoS One. 2017 Feb 9;12(2):e0171753. doi: 10.1371/journal.pone.0171753 (PMC5300176; doi:10.1371/journal.pone.0171753)

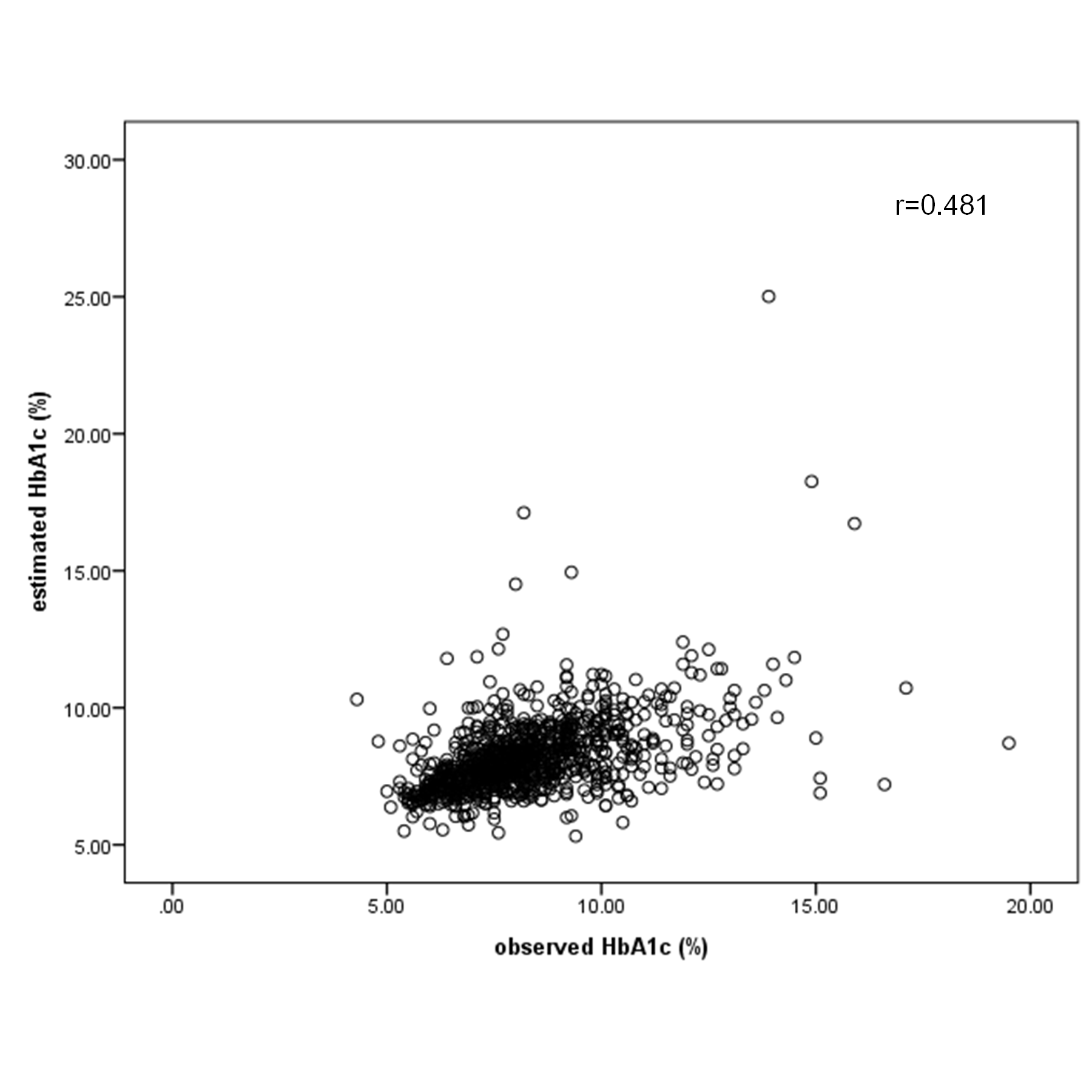

Supplement: S1 Fig — (TIF) [file pone.0171753.s001.tif]

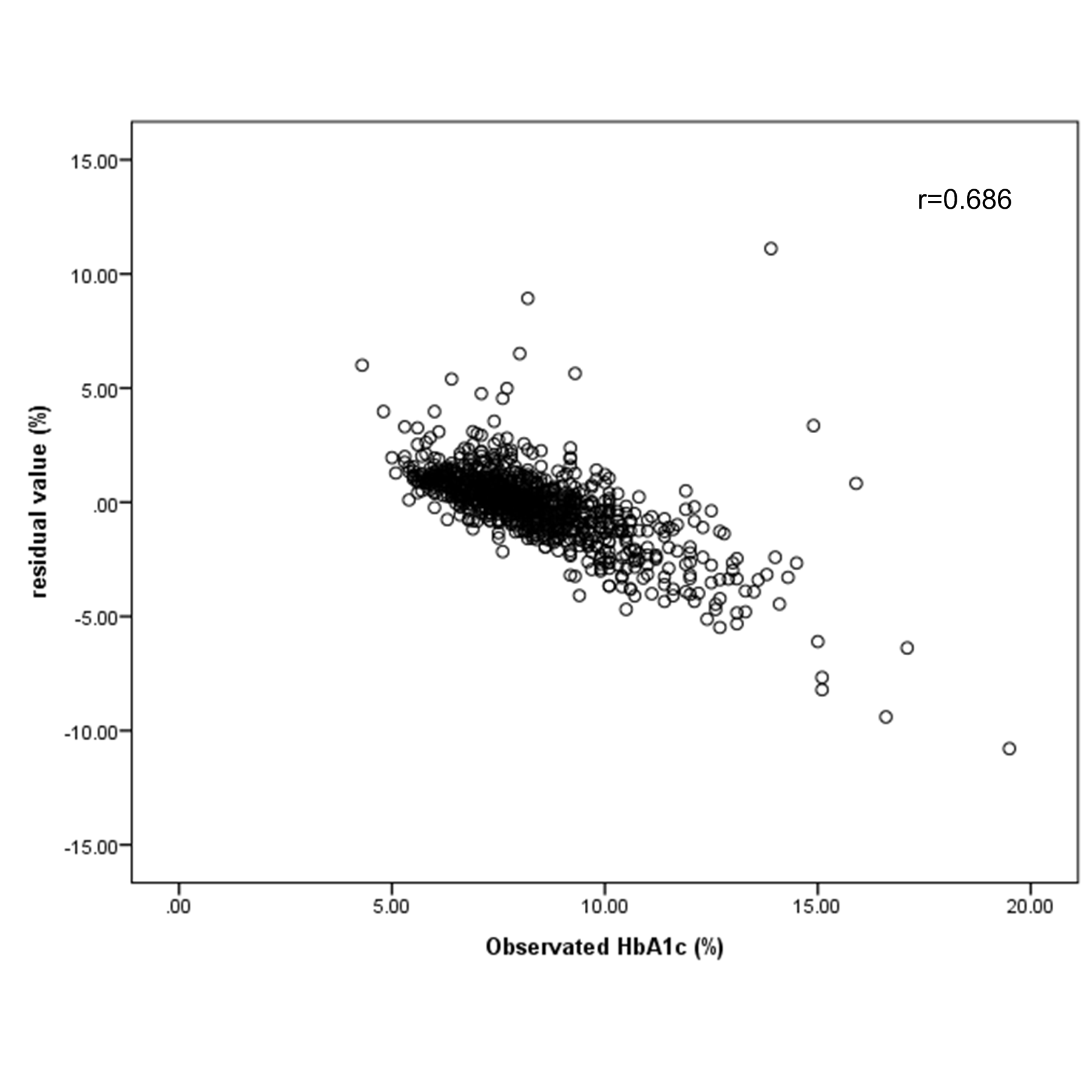

Supplement: S2 Fig — Residual value = estimated value of HbA1c (%) minus observed value of HbA1c (%) (TIF) [file pone.0171753.s002.tif]
